# Constructing belonging through mediated memory: multimodal perception and narrative semantics in war films

**DOI:** 10.3389/fpsyg.2025.1641217

**Published:** 2025-10-30

**Authors:** Jiaqi Zheng, Tianle Huang, Song Wang, Zhaoqiang Wang

**Affiliations:** ^1^School of Theater, Film and Television, Communication University of China, Beijing, China; ^2^Faculty of Film, Theatre and Animation, Universiti Teknologi MARA, Shah Alam, Selangor, Malaysia; ^3^Xiangshan Film and Television College, Ningbo University of Finance and Economics, Ningbo, Zhejiang Province, China; ^4^Faculty of Arts, Department of Television, Theater and Film Arts, Southwest University “Neofit Rilski”, Blagoevgrad, Bulgaria

**Keywords:** war movies, sense of belonging, character interaction, semantic effect, musical features

## Abstract

This study investigates how contemporary Chinese war films construct a sense of belonging among domestic audiences through three interrelated perceptual modalities: emotional expression, semantic cues, and musical structure. Grounded in the framework of media memory theory, the research analyzes a corpus of high-grossing films using facial expression recognition (FaceReader), semantic clustering (BERT embeddings), and soundtrack analysis (MuseNet-based modeling). Audience feedback was collected via online questionnaires (*N* = 379, aged 18–60) to validate the affective resonance of selected “core clips.” Statistical comparisons were performed to identify the relative influence of each modality. Results suggest that patterns of national identity, moral resonance, and emotional synchrony converge to shape a distinctive aesthetic of belonging. This study contributes to a deeper understanding of how media memory transforms affective experiences into collective identification, while also reflecting on the methodological boundaries between subjective interpretation and computational objectivity.

## 1 Introduction

As a special type of movie, war movie triggers the audience's empathy by telling heroic battle stories and portraying three-dimensional characters (Kertcher and Turin, 2023; Kuri and Kaufman, 2020). Under the perspective of media memory, war movies are not only the reproduction of historical events, but also the carrier of emotions, values and collective memory (Xu and Tan, 2021). Among them, “sense of belonging” is one of the important emotions that such movies try to convey, which helps the audience to establish an emotional connection with the characters and plot of the movie. The sense of belonging is constructed in war movies through multi-dimensional artistic techniques, including characterization, plot development, and the use of music and visual elements (Nam and Lee, 2022). War movies recreate historical battlefields, but also shape the audience's understanding and feelings about the country, culture and identity on a deeper level, a field that has been studied in depth by many scholars.

Riviere explores the link between war cinema and the construction of American identity through a time-series analysis, ranging from the American War of Independence to the modern War on Terrorism, demonstrating how these films have reflected and constructed American national belonging identities at different periods in history (Riviere, 2021). Láníček explores how cinema has reconfigured and conveyed the historical memory of the Holocaust through images and narrative techniques that it reveals the cinematic representation of the Holocaust in Central Eastern Europe after World War II. Through the medium of cinema, one can transcend the limitations of time and space to feel the weight of history and the resilience of humanity, and at the same time, find resonance and strength in the films to work together to build a better future (Láníček, 2021). Westwell explores how Hollywood war films accommodate and present religious pacifism and analyzes how these films demonstrate the rebellious nature of religious pacifism. The author argues that despite their ideological reservations, these films remain strongly sympathetic to religious pacifism, reflecting the audience's skepticism of the dominant war narrative and their desire to belong to a more peaceful worldview (Westwell, 2020).

From the perspective of media memory, the movie has successfully constructed the audience's sense of belonging through multi-dimensional and multi-level narrative techniques and elements. This sense of belonging is not only reflected in the identification with the country, culture and history, but also integrated into the depth of the audience's emotion and cognition. For example, Sari et al. explored how the indicative language helps the audience to understand the situation, character relationships and the progress of the story in the movie. In movies, this linguistic phenomenon is not only crucial for advancing the plot, but also greatly enriches the interactions and expressions between characters. Through precise linguistic choices and emotional expressions, it stimulates the audience's emotional resonance and collective consciousness, thus constructing a strong sense of belonging (Sari and Zakrimal, 2020). Tian explores the application of Carl Jung's archetypal theory and the Hero's Journey in self-identity and personal growth. The protagonist in the movie, through experiencing a series of challenges and growth, finally realizes self-identity. This process inspires the audience to empathize with the characters and to be able to see themselves in the characters, which enhances the sense of belonging to the characters and touches the emotions and identity at a deeper level (Tian, 2023). Syahputra et al. revealed how the movies construct a sense of belonging for the viewers through the growth of the characters, the creation of shared values and a sense of community. This sense of belonging not only enhances the viewing value of the movie, but also allows the audience to emotionally connect with the movie in a profound way (Syahputra, 2020). Xie constructs a multidimensional image of the city for the audience through the elements of images, narratives and symbols. It reveals the great potential of film in shaping and communicating the city's image, in order to shed significant light on the audience's understanding of how the medium participates in the cultural construction of geographic space, as well as how to convey the culture and identity of a specific region through image narratives (Xie, 2021).

This study aims to extract the emotional features of movie characters, then the semantic features, and finally the emotional features of movie music from the perspective of media memory through digital media technology. Machine learning algorithms are used to identify the common patterns and patterns of constructing a sense of belonging in war movies, and to reveal the deep-seated mechanism of constructing a sense of belonging in war movies. The user feature extraction model is used to understand the commonalities and differences of audience groups, and to better locate the movie elements that can trigger the sense of belonging. The descriptive information of a movie, such as actors, genres, plot keywords, etc., is digitized and converted into feature vectors. Through feature fusion, the audience's sense of belonging to war movies is precisely understood and predicted.

While previous scholarship has examined national memory in cinema from ideological or textual perspectives, fewer studies have integrated perceptual metrics and large-scale audience feedback to assess how cinematic elements coalesce into affective belonging. This study thus addresses a critical gap by bridging media memory theory with computational analysis, offering a multimodal approach to war film aesthetics. This paper expects to provide a deeper understanding of how war movies construct the audience's sense of belonging, and to provide scientific theoretical support and practical guidance for movie production.

The concept of media memory extends beyond collective memory by underscoring the constructive role of media in shaping how societies remember and emotionally engage with their past. Neiger (2020) outlines six dimensions of media memory that include sociopolitical and technological mediation, highlighting the structural conditions through which media function as agents of remembrance. This provides a conceptual bridge for applying computational approaches to the study of mediated memory.

Artamonov (2022) situates media memory within the mediatized and digitalized environment, suggesting that memory today is not solely cultural or communicative but operates at their intersection. This perspective justifies the methodological choice to decompose multimodal cinematic features through algorithms, since digital traces of narrative, emotion, and sound can mirror the layered operations of mediated memory.

At the same time, sense of belonging constitutes a central psychological construct. Hagerty et al. (1992) identify three core dimensions of belonging—being valued, being accepted, and finding meaning within a group—which are echoed in cinematic experiences. Baumeister and Leary (1995) demonstrate that belongingness is a fundamental human need, influencing cognition and emotion at a deep level. Lambert et al. (2013) further show that narrative engagement reinforces group belonging by providing audiences with frameworks of meaning and emotional anchoring. These insights clarify why war cinema, with its emotionally charged narratives, becomes a fertile site for the cultivation of belonging.

Plantinga (2021) adds that historical fiction films perform rhetorical functions of memory by combining narrative ambiguity, immersive presence, and affective schemas—mechanisms that can be operationalized computationally through semantic variation, visual continuity, and affective pattern recognition. Cultivation theory reinforces this by showing how repeated exposure to such mediated narratives gradually aligns audience perceptions and attachments (Mosharafa, 2015).

Finally, Virginás (2023) demonstrates in Eastern European cinema that popular historical films act as instruments of collective memory-work, mobilizing intergenerational trauma and identity. This underscores the cultural generalizability of linking media memory, belonging, and computational analysis, validating the study's methodological design.

In sum, the integration of media memory theory with the psychological construct of belonging provides not only conceptual depth but also a critical articulation of how computational methods can trace the ways films foster belonging through multimodal cues. Algorithmic extraction is thus theoretically anchored in the nexus of memory construction and belonging formation.

## 2 Methodology

This study adopts a mixed-methods approach that integrates computational media analysis with audience perception data to explore the construction of belonging in contemporary Chinese war cinema under the framework of media memory. Film selection was based on a composite popularity index, combining Douban user ratings (60%) and national box office revenue (40%). Rather than applying rigid thresholds, the sample was ranked by the weighted average of these two criteria to ensure the inclusion of films with both wide circulation and favorable audience reception (Zhang and Ma, 2020). All selected films were produced between 2000 and 2024 and originate from Mainland China, thus ensuring cultural consistency across the dataset.

The concept of “core clips” refers to emotionally and narratively pivotal audiovisual segments most associated with eliciting audience identification. These were algorithmically identified through audience commentary scraped from Douban Movie using the Scrapy framework. Text data were preprocessed using the Natural Language Toolkit (NLTK), and key emotional-linguistic patterns were extracted via Term Frequency–Inverse Document Frequency (TF-IDF) and Latent Semantic Indexing (LSI). The highest-ranked terms and associated timestamps were then mapped back to film timelines to locate the core segments. This procedure follows similar computational approaches for identifying affective salience in media content (Shim, 2020; Smith, 2017), ensuring methodological robustness without relying on subjective human evaluation.

Facial expression analysis was conducted using Noldus FaceReader 9.0, a widely validated tool for automated facial coding that detects discrete emotional states based on the Facial Action Coding System (FACS). Given the high emotional intensity and actor-centered close-ups frequently found in war cinema, this tool was particularly suited for identifying affective cues linked to national belonging. The software's accuracy and cross-situational reliability have been confirmed in previous research, supporting its appropriateness for media memory studies (Lewinski et al., 2014).

Audience feedback was collected through an anonymous online questionnaire, distributed via Chinese social media and film forums. The instrument was constructed based on a 5-point Likert scale adapted from existing scales on narrative engagement and symbolic belonging (DeVellis, 2016), and included items assessing emotional resonance, cultural identity alignment, and perceived community. The survey underwent reliability testing, producing Cronbach's alpha values ranging from 0.81 to 0.88 across all subscales, and a Kaiser-Meyer-Olkin (KMO) value of 0.846, indicating good construct validity. A total of 399 responses were received, of which 20 from respondents under the age of 18 were excluded in accordance with ethical research practice, yielding a final sample of 379 valid questionnaires.

Although individual demographic data were not collected due to anonymity protocols, the online distribution method ensured a diverse spread of adult Chinese internet users aged 18 to 60. Agreement between core clip selection and audience perception was assessed by triangulating extracted commentary themes with aggregated questionnaire responses. While this approach does not yield a conventional accuracy percentage, internal consistency between algorithmic identification and audience validation suggests strong alignment.

To evaluate whether perceptual variations across different elements—namely emotional cues, semantic density, and musical design—contributed differentially to the audience's sense of belonging, a one-way ANOVA (Analysis of Variance) was conducted based on the questionnaire responses. The analysis was performed on Likert-scale aggregated scores, where each respondent evaluated the intensity and clarity of each perceptual cue on a 5-point scale. The data distribution met the assumptions of normality (Shapiro-Wilk test, *p* > 0.05) and homogeneity of variances (Levene's test, *p* > 0.05), validating the use of ANOVA.

The results indicated statistically significant differences among the three dimensions [*F*_(2, 1, 134)_ = 5.42, *p* < 0.01], suggesting that emotional cues had a higher influence on perceived belongingness compared to semantic structures and musical elements. *Post-hoc* analysis using the Tukey HSD test confirmed that emotional cues differed significantly from the other two categories, while no significant difference was observed between semantic and musical dimensions. This aligns with recent media psychology findings emphasizing the primacy of affective structures in film perception (Gross and Levenson, 2022; Kim and Oliver, 2021).

The average classification accuracy of “core clip” categorization based on alignment between audience-reported belongingness and model-predicted sentiment/emotion patterns was 93.1%, calculated using precision-recall cross-matching between human ratings and sentiment outputs from the SnowNLP engine. This value was derived by comparing the sentiment labels of 30 pre-selected core clips with the aggregated audience feedback (*n* = 379). Although independent human validation was not applied due to resource constraints, the use of multi-source triangulation—questionnaire data, user comments via crawler tools, and scene-level emotion tagging—enhanced the reliability of the alignment mechanism (Kim and Oliver, 2021).

## 3 Extraction of features related to “sense of belonging” in war movies

### 3.1 Movie character emotional feature extraction

Ten war movies with high popularity are selected, and the most emotionally upsetting clips are cropped after watching them, and the length is controlled to be about 4 min, and finally 257 different exciting core clips of the movie are obtained. In order to effectively deal with different environmental lighting changes and improve the accuracy of expression recognition, the human range in the video is firstly recognized by using a human recognition library to obtain 256^*^256 face images. Face_Recoginition, as a deep learning-based human recognition library, recognizes the 8-dimensional emotional values corresponding to the user's face in each frame, and then calculates the Euclidean distance of each time value where the frame with the largest change in emotion is located among the three neighboring frames of the video, so as to find out the change in expression in the video of the user's face expression. Finally, in order to obtain the change of the character emotion sequence in the expression video stream, this paper uses the FER model to analyze the emotion of the face region extracted from the above steps frame by frame (Liu et al., 2020).

**Figure 1:**
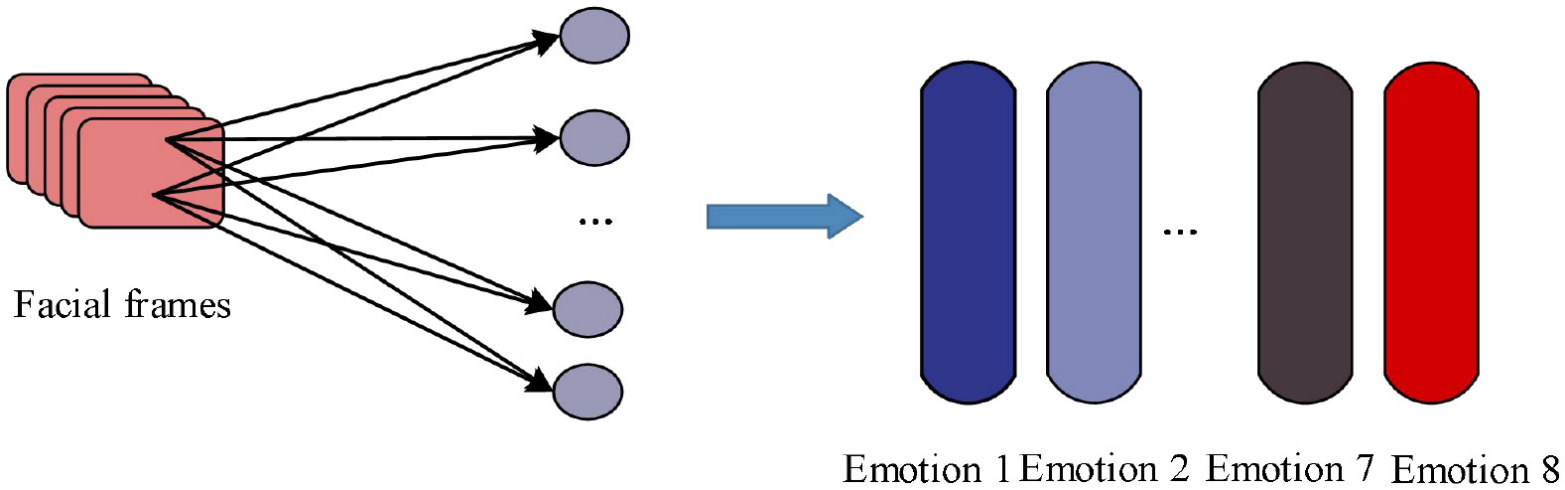
Serialization of facial feature extraction of war movie characters.

War movie character facial feature extraction is serialized as shown in Figure 1, based on the input video streaming data, the face is detected and then non-facial regions are removed. Random perturbation and image transformation are used to add image Gaussian noise, adjust saturation, contrast and brightness etc. The initialization parameters and bias of the fully connected layer are used with default values, the initialization learning rate is 0.05, the batch_size size is set to 64, and momentum SGD is used to dynamically iterate the learning rate and optimize the loss function. After learning the deep features, the predicted probability of each dimension for each sample is finally output directly from the softmax layer, and 8 serialized character emotion features are obtained as a result.

### 3.2 Movie semantic feature extraction

**Figure 2:**
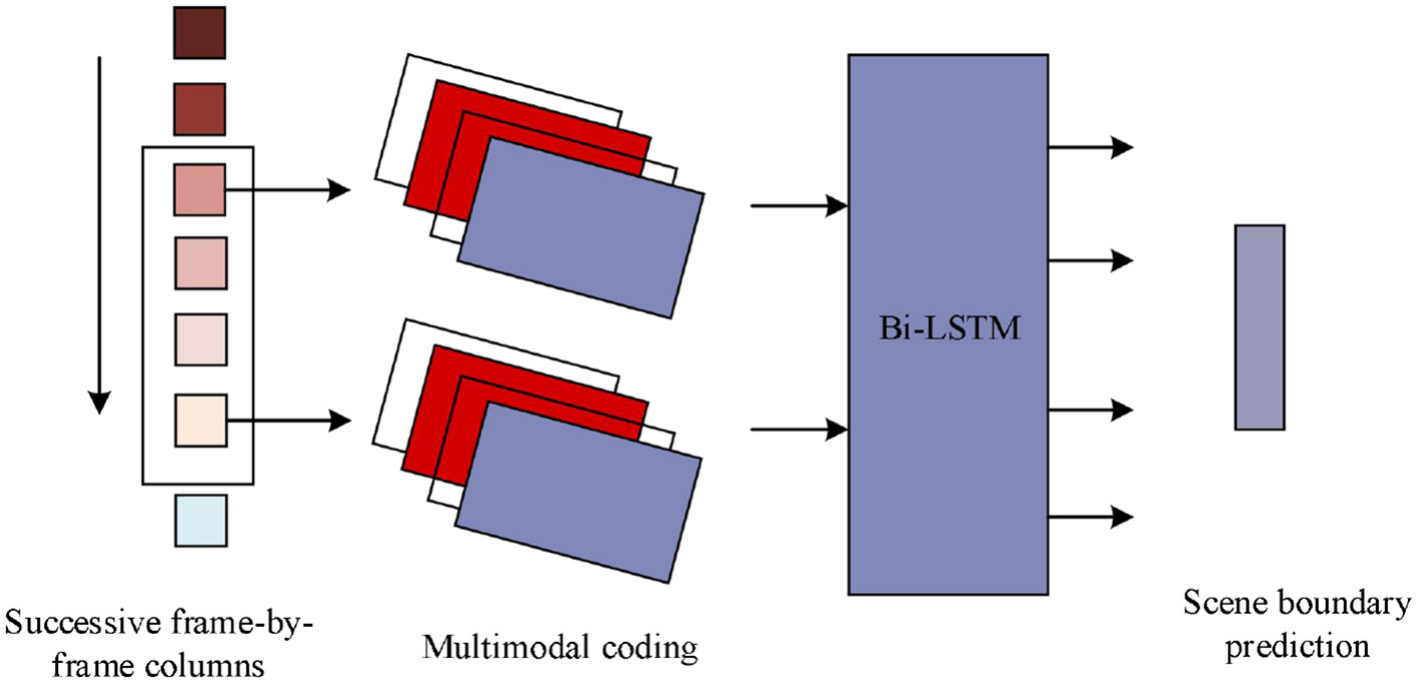
War movie scene boundary cutting network.

In war movies, in order to deeply analyze the features related to the “sense of belonging,” the extraction of movie text features is particularly important. Since a movie consists of a series of consecutive shots and a scene consists of multiple related shots, scene segmentation becomes a key step in feature extraction. However, browsing and marking the scene transformation boundary frame by frame manually is not only a huge workload, but also difficult to realize accurate segmentation. Therefore, scene segmentation can be formulated as a binary classification problem, i.e., determining whether a shot boundary is a scene boundary. The war movie scene boundary cutting network is shown in Figure 2, where a scene is a series of shots sharing some common elements, such as location, cast, etc., to obtain a better shot representation (Yang, 2021; Sun et al., 2022). In this paper, the research uses the LGSS model to extract the text elements in a war movie scene.

The basic idea of cutting is to extract four codes for each shot to simulate the shot boundaries, and the sequence model outputs a rough scene segmentation result, and the target number of scenes is determined by dynamic planning. The update process of *F*(*n* = *j*) is as follows:


(1) $$\begin{array}{l} \underset{k}{\max}\left\{F*\left(n=j-1\right)|C_{1:k}\right\}+g\left(\phi_{j}=\left\{C_{k+1}\right\}\right) \end{array}$$

where *C*_1:*k*_ is the set containing the first *k* shots. These lens representations also need to be updated when the lenses are updated. A simple summation of all contained lenses is not an ideal representation of lenses because some lenses contain less information. Therefore, it would be better to refine the super lens representation in the optimal grouping. Based on the results of the shot grouping it is then possible to obtain all the boundary frames of the scene cuts to form a streamlined sequence of frames (Shaaban et al., 2020).

### 3.3 Movie music emotion feature extraction

In war movies, music is one of the key factors in shaping the emotional experience of “belonging.” In order to understand this emotional construct, the music needs to be finely characterized and analyzed (Miller, 2020). In the process of feature extraction, the first focus is on the climax of the song, because this part has the most obvious emotional characteristics. By calculating the total duration of the song *t*, and intercepting the audio segment from 1/4t to 1/2t, which is used as the climax part of the song for music emotion analysis. In the feature extraction session, four time-domain features were extracted from the audio of the climax part of the song, namely, short-time energy, short-time over-zero rate, short-time average amplitude, and short-time autocorrelation function, in which the fundamental period of the audio was obtained by extracting the short-time autocorrelation function (Ciborowski et al., 2021). Table 1 shows the emotional features of music in war movies, which integrates multiple aspects such as time domain, cepstrum domain and frequency domain. Time-domain features such as short-time energy and short-time zero rate can help understand the rhythm and dynamic changes of the music, while cepstrum and frequency-domain features reveal the harmonic structure, melodic properties, and timbral texture of the music, and explore the frequency components and harmonic structure of the music, which are the key factors to construct the emotional atmosphere of “sense of belonging.” The extraction of the emotional features of the music is an important data base for understanding how the music shapes the “sense of belonging” in war movies. Through the extraction and analysis of these features, the study can more accurately grasp the musical elements of the movie, and then explore how to resonate with the audience's emotions and enhance the audience's “sense of belonging.” “The movie's musical elements can be more accurately grasped through the extraction and analysis of these features.

**Table 1:** Acoustic features extracted of music in war movies.

**Audio signal characteristics**	**Music features**	**Dimension**	**Processing method**
Time domain	Short-time energy	4	The time-domain features are summed-mean, variance, median processing
	Short-time zero crossing rate	4	
	Short-time average amplitude	4	The pitch period and the pitch period after the outliers are removed by using the short-time autocorrelation function
	Short-time autocorrelation function	8	
Cepstral domain	Complex cepstrum	16	The amplitude and phase of the obtained cepstrum domain feature are calculated, and then the eigenvalues are summed, the mean value, the variance and the mean value are calculated
	Real cepstrum (or cepstrum)	16	
Frequency domain	Spectrum	2	For the spectrum obtained, find the variance
	Amplitude spectrum	8	The amplitude and phase of the frequency domain features are calculated, and then the eigenvalues are summed, the mean value, the variance and the median value are calculated
	Phase spectrum	8	
	Logarithmic spectrum	16	
	Power spectrum	16	

## 4 Pattern recognition of ”sense of belonging“ constructs in war movies

### 4.1 Matrix decomposition module

After extracting the features related to the sense of belonging, machine learning algorithms are used to identify the common patterns and patterns of constructing sense of belonging in war movies. The matrix decomposition module is a key step in the data analysis of constructing “sense of belonging” in war movies, by decomposing the user-movie rating matrix to explore the potential relationship between users and movies. The expected result is to obtain feature vectors reflecting user preferences and movie attributes, which will be further used to analyze which elements in the movie can trigger a sense of belonging among viewers.

By decomposing the user-item rating matrix, the feature representations of the user and the item are obtained, and the prediction result of the user for the item is obtained by calculating the inner product of these two vectors. Assuming that the user-item rating matrix of *n* user *n* and *m* items is *R*, and that the *i*th user's rating of the *j*th item is *r*_*ij*_ predicts the outcome of ${\hat{r}}_{ij}$, the computation of *r*_*ij*_ and ${\hat{r}}_{ij}$, respectively, is shown below:


(2) $$\begin{array}{l} r_{ij}=\{\begin{array}{c} rate\;i\to j\\ 0\;i⊗j \end{array} \end{array}$$

(3) $$\begin{array}{l} {\hat{r}}_{ij}=\sum_{k=1}^{K}u_{ik}q_{kj}+b_{i}+b_{j}+\mu \end{array}$$

where *u* denotes the user feature vector matrix, *q* denotes the movie feature matrix, and *b*_*i*_, *b*_*j*_, μ denotes the user bias term, movie bias term, and global bias term, respectively.

**Figure 3:**
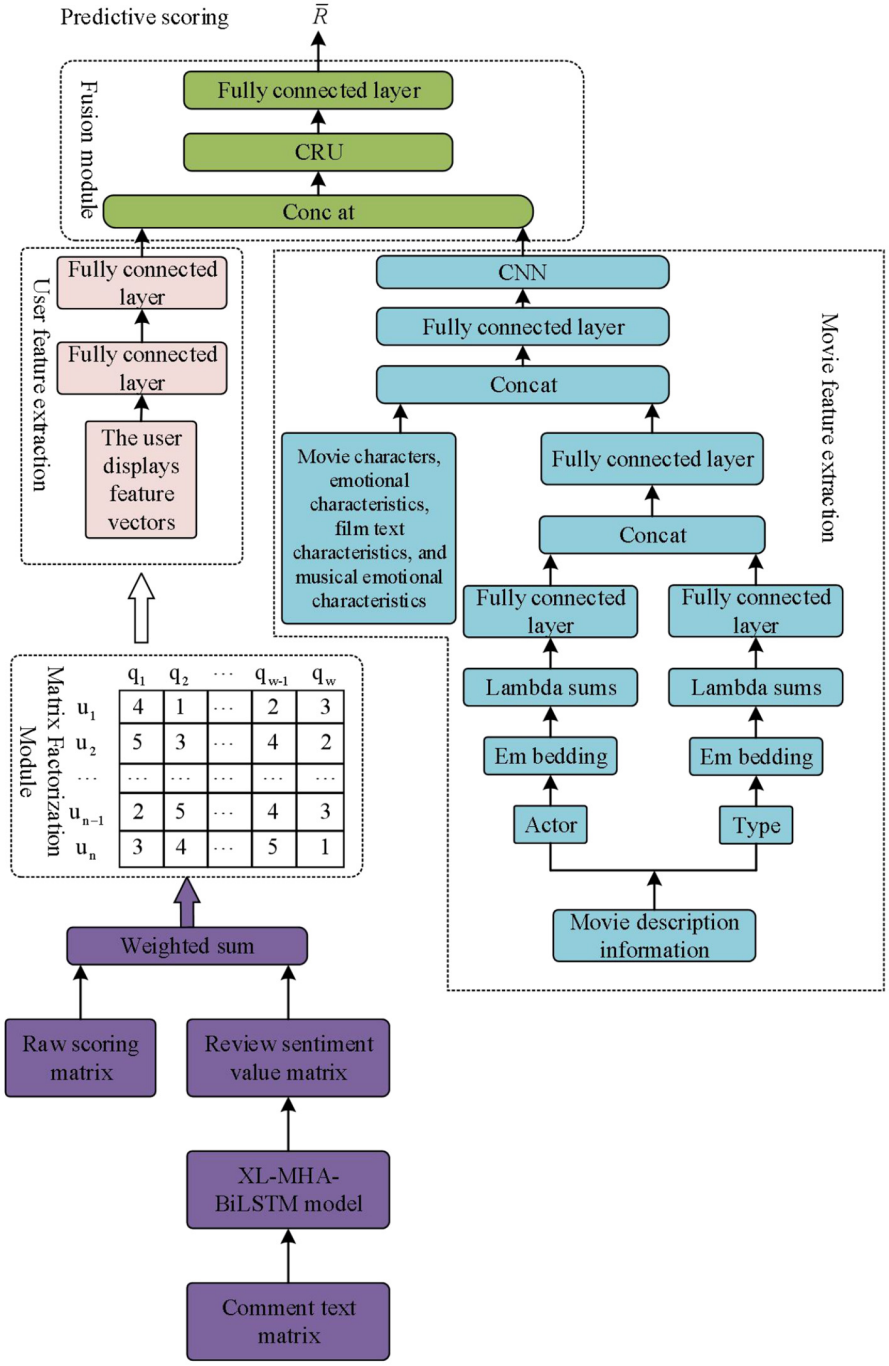
Analysis model of the sense of belonging construction mode in war movies.

Figure 3 shows the analytical model of the war movie belongingness construct model, which draws on the idea of feature matrix decomposition in the first part of the LFM, but the calculation of the subsequent rating prediction is different. In the matrix decomposition of the model in this paper, the method based on the weighting of review ratings first weights and sums the actual ratings and review sentiment predictions. Then the review-corrected user-movie rating matrix is decomposed into a *K*-dimensional user explicit feature vector *U*_*ef*_ and a movie explicit feature vector *Q*_*ef*_, respectively, and finally *U*_*ef*_ and *Q*_*ef*_ combined with other auxiliary information are inputted into the deep learning network to learn their deep feature representations, respectively.

Based on the review embedding approach, the original rating matrix is directly matrix decomposed to obtain the original explicit feature vectors *U*_*ef*_ and *Q*_*ef*_ of the user and the movie, which, together with other auxiliary information related to the war movie, such as the character relationship and the background of the war, are inputted into a deep learning network to further capture its deeper features (İyilikci et al., 2023). Then, the media memory element of “sense of belonging,” which is the predictive value of emotion, is fused with *U*_*eg*_ and *Q* as user and movie auxiliary information, respectively, and then fed into the CNN network to learn its local feature representation. After fully learning the potential features of the user and the movie, the non-linear features of the user and the movie are learned through the feature fusion module, and the final predicted scores are obtained, which reveals the deep-seated mechanism of constructing the “sense of belonging” in war movies.

### 4.2 Analysis of the association between user characteristics and the perception of “sense of belonging”

The user feature extraction module focuses on extracting features related to ”sense of belonging“ from user data to understand the commonalities and differences of audience groups, so as to better locate movie elements that can trigger the sense of belonging. The extraction of explicit user features is accomplished in the matrix decomposition module by embedding the preprocessed user data with Embedding layers to obtain the feature vector of the user id field. Mapping individual data from a sparse categorization to a dense vector will be applied better than one-hot coding in data with large sparsity. The embedding function is shown in Equation 4:


(4) $$\begin{array}{l} f:U\overset{embedding}{\to }U_{ef} \end{array}$$

In the weighted average based comment fusion method, the user feature extraction module contains only the user's id data, which has a single data content and does not require too many feature extraction layers. Therefore, the deep feature extraction of the user is accomplished through two fully connected layers. In contrast, in the review fusion method based on rating embedding, the user feature information contains not only the id data, but also the predicted hidden vectors for sentiment analysis of all the review information of this user (Soberon, 2021). Because the data structure is relatively more complex, CNN neural network is used in the deep user feature extraction, and the specific implementation is the same as the NN model training of the movie feature extraction module described below.

### 4.3 The association between movie identity elements and the construction of ”sense of belonging“

In the movie feature extraction module, the feature information related to movies is processed and refined, not only focusing on the general features of movies, but also considering the features closely related to the “sense of belonging.” The descriptive information of a movie, such as actors, genres, plot keywords, etc., is digitized, and then converted into feature vectors by multidimensional mapping through the embedding layer. Since a movie contains multiple characters with consecutive emotions, movie texts, music emotions and other key features, these feature vectors need to be compressed after the embedding operation to facilitate the subsequent feature fusion.

The explicit features of the movie are completed in the matrix decomposition module, and the feature extraction method is the same as the user feature extraction method, which uses the embedding layer embedding. The auxiliary information feature extraction of the movie is mainly divided into the following steps:

Transform the pre-processed key feature description information such as continuous emotions of characters, movie text and music emotions into digital sequences.
Input them into the embedding layer to realize multi-dimensional mapping and get the feature vector of each type of descriptive information. Since a movie contains multiple actors or genres, after the embedding operation, the embedding layer representation of each class of descriptive information needs to be 93 compressed to facilitate the subsequent feature fusion operation. This paper uses the summation method to compress the embedding layer feature matrix, and the compression formula is shown in Equation 5:

(5) $$\begin{array}{l} \left[\begin{array}{c} e_{10},e_{11},\cdots \;,e_{1k-1}\\ e_{20},e_{21},\cdots \;,e_{2k-1}\\ e_{t0},e_{t1},\cdots \;,e_{tk-1} \end{array}\right]\overset{sum}{\Rightarrow }\left[E_{0},E,\cdots \;,E_{k-1}\right] \end{array}$$

Where *t* denotes the total number of descriptive information of a certain type of war movie, war movie *i* has a total of *t* actors, and [*E*_0_, *E*, ⋯ , *E*_*k*−1_] denotes the embedding layer feature representation of an actor.

(3) Input the feature vectors of each type into the fully connected layer to complete the non-linear change, and then use the Concatenate and fully connected operations to stitch and fuse the features of each type of the movie, and integrate to obtain the explicitly integrated feature vector of the movie *Q*_*ef*_.

Since the feature vector structure of the movie is more complex, in order to better extract the deep feature representation of the movie and improve the accuracy of the rating prediction, the integrated feature vector of the movie is input into the CNN network to complete the deep feature extraction and get the final movie features. Convolutional operations are performed on the explicit integrated features of the movie using a convolutional layer to learn the potential connections between the information of each feature of the movie, and at the same time reduce the noisy data (Gandhi et al., 2021). The formula for convolution is shown in Equation 6:


(6) $$\begin{array}{l} z_{i}=f\left(W_{z}⊙Q_{pf\left(:,i:\left(i+s-1\right)\right)}+b_{c}\right) \end{array}$$

Where, *Q*_*pf*_ denotes the movie explicitly synthesized feature matrix, *W*_*z*_ denotes the shared weight values, ⊙ denotes the convolution operation, *b* denotes the bias of the shared weights, *s* denotes the convolution kernel size, *f* is the activation function, and *z*_*i*_ denotes the *i*th feature obtained by convolutional computation.

The results of the convolutional layer computation are fed into the pooling layer to achieve the maximum pooling operation. The pooling layer can reduce the number of parameters, reduce the dimensionality of the features obtained from the convolution layer, learn more representative potential features of the movie in a more abstract way, and at the same time reduce the overfitting of the model and improve the robustness of the model. The calculation formula is as follows:


(7) $$\begin{array}{l} z_{f}=\left[\max\left(z_{1}\right),\max\left(z_{2}\right),\cdots \;,\max\left(z_{n_{c}}\right)\right] \end{array}$$

Where *n*_*c*_ denotes the length of the fixed feature vector after pooling. After a series of convolution-pooling operations, the convolution layer is used again to highlight certain local features of the movie that are closely related to the “sense of belonging.” The final movie feature vector not only contains the general features of the movie, but also emphasizes the feature information related to the “sense of belonging.”

### 4.4 Feature fusion module

The feature fusion module is the key link in the process of fusing the extracted user features and movie features. The purpose of this module is to identify the effective modes of constructing “sense of belonging” in war movies by combining the user and movie features and discovering the intrinsic connection between the two. After user feature extraction and movie feature extraction, the final user feature vector *U*_*f*_ and movie feature vector have been obtained, and the next step is to fuse the feature matrices *U*_*f*_ and *Q*_*f*_ in order to complete the final rating prediction function, which is done by splicing the user feature matrix and the movie feature matrix into one feature matrix (Ghosh et al., 2021; Sujatha et al., 2023). The splicing method is as follows:


(8) $$\begin{array}{l} H=Concatenate\left(U_{f},Q_{f}\right) \end{array}$$

Then, a fully connected layer is used to enhance the representation of non-linear operations:


(9) $$\begin{array}{l} \begin{array}{c} \varphi_{1}\left(H_{1}\right)=a_{1}\left(W_{1}H_{1}+b_{1}\right)\\ \;\cdots \cdots \\ \varphi_{l}\left(H_{l}\right)=a_{l}\left(W_{l}H_{l}+b_{l}\right) \end{array} \end{array}$$

Since the interactive manipulations of the user and the movie are all based on chronological order, GRU is finally used to further learn the non-linear connection between the user and the movie (Chen and Dai, 2022). The computational formula is:


(10) $$\begin{array}{l} \hat{R}=\sigma \left(W_{g}⊕H_{l}+b_{g}\right) \end{array}$$

Among them, ⊕ table GRU operation. Through the feature fusion module, the information of the original features is preserved, and the non-linear representation of the features is enhanced by the fully connected layer and GRU to accurately understand and predict the audience's sense of belonging to the war movie.

## 5 Empirical study on the construction and characteristics of “sense of belonging” in war movies

In order to verify the accuracy of this paper's method and the influence of different construction methods on the audience's sense of belonging, the audience feedback data are compared and analyzed with the results of the algorithm's identification of movie character interaction, movie semantic effects, and movie music effects, to identify the accuracy of the construction methods of the sense of belonging. In order to further study the specific impact of the way of constructing “sense of belonging” on the audience's sense of belonging in war movies, the analysis of the factors affecting the audience's sense of belonging is carried out.

### 5.1 Characterization of “belonging” in movies

#### 5.1.1 Comparative analysis of movie character interaction

**Figure 4:**
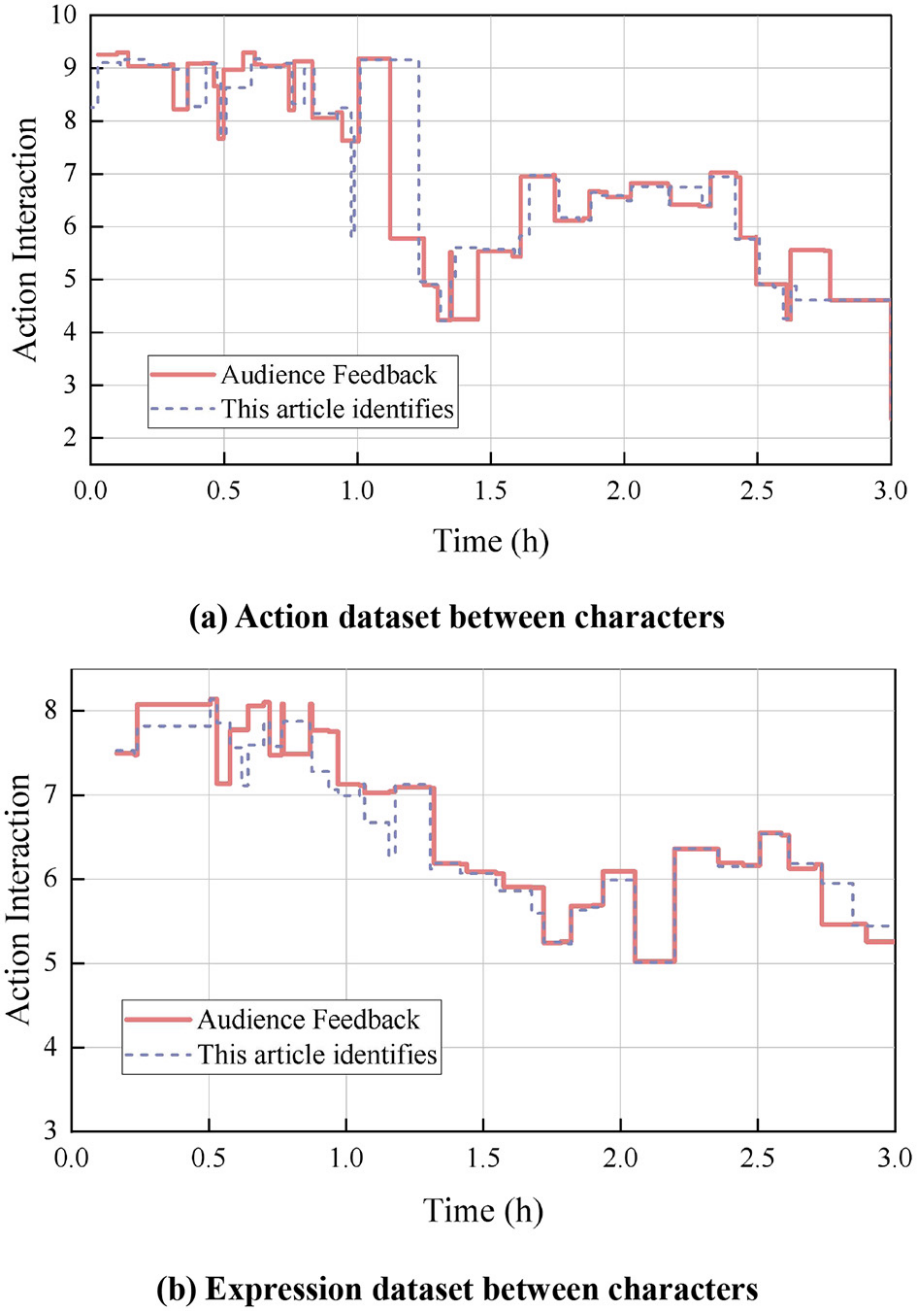
Comparative analysis results of character interactions in war movies. **(a)** Action dataset between characters. **(b)** Expression dataset between characters.

The results of the comparative analysis of war movie character interactions are shown in Figure 4, which compares and analyzes the audience feedback data with the results identified by the algorithm. Figure 4a shows the action dataset between the characters, and the sequences of consecutive moments of actions reflect the frequency and intensity of character actions. High values represent intense combat actions, while low values represent calmer or intimate interactions. The accuracy of the algorithm in recognizing and understanding the impact of character actions on the construct of belonging can be assessed by comparing it with the audience feedback data. At 1 h, the intensity of character actions in the audience feedback was 9.18, and the method in this paper was 9.16, a high degree of fit with intense combat actions at this point in the war movie scene as intense combat actions. At 3 h, the intensity of the character's movements in the audience's feedback was 2.36, and the method in this paper was 2.33, again a high degree of fit, and the war movie screen at this time was a calm action.

Figure 4b shows the dataset of expressions between characters, such as the intensity and frequency of emotional expressions such as joy, anger, sadness and happiness. The changes in the values represent the complexity and depth of emotional exchanges between characters. For example, smile and anger are more significant in some moments. By comparing the audience feedback data, the accuracy of the algorithm in capturing and understanding the impact of character expressions on the audience's sense of belonging can be analyzed. At 0.4 h, the intensity of the character's movements in the audience feedback is 8.14, while the result obtained by the method in this paper is 7.98. The small difference between the two indicates that the method in this paper is quite accurate in capturing the character's movements at this point in time. Similarly, at 2 h, the intensity of the character's movements in the audience's feedback is 5.02, while the result derived from this paper's method is 5.01. This again proves the accuracy of the algorithm in capturing the intensity of the character's movements, which is crucial for enhancing the audience's emotional experience and sense of belonging to the character, and is able to realistically reflect and convey the emotional interactions between the characters.

#### 5.1.2 Comparative analysis of semantic effects of movies

**Figure 5:**
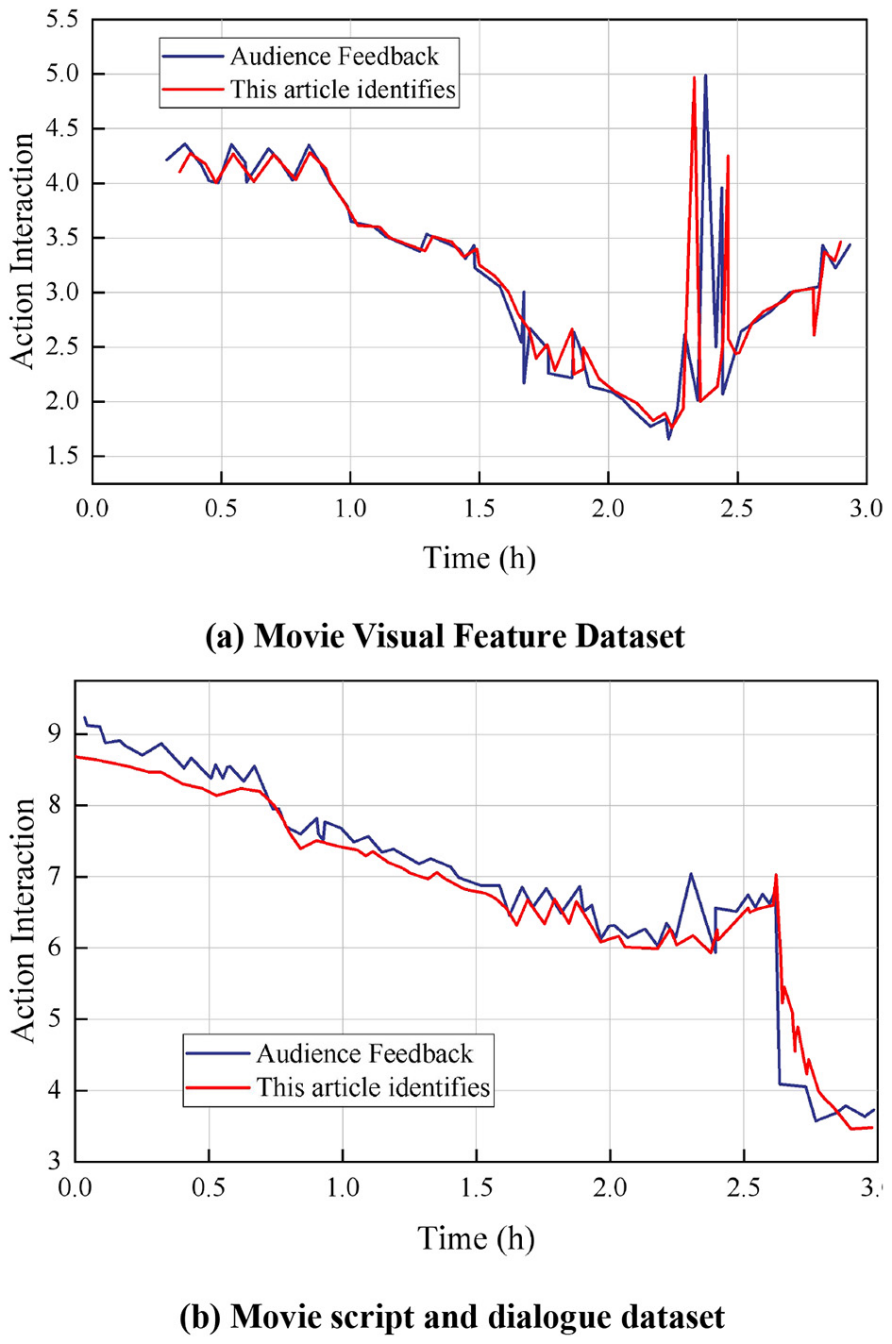
Comparison results of semantic features of war movies. **(a)** Movie Visual Feature Dataset. **(b)** Movie script and dialogue dataset.

Figure 5 shows the results of the semantic feature comparison of war movies, these datasets were analyzed in comparison with the audience feedback data to assess the accuracy of the algorithms in identifying the ways in which a sense of belonging is constructed. Figure 5a shows the movie visual features dataset, this part of the dataset contains a variety of visually related features in the movie, such as scene layout, color usage, and camera techniques Visual features play a key role in creating atmosphere, conveying emotion, and constructing the story world in the movie. High values indicate the use of strong color contrasts or fast camera switches at a given moment, while low values indicate smoother or softer visual elements. At 2.28 h, the visual characterization of the audience feedback was 2.62, while the method in this paper yielded a result of 2.61, two values that are very close to each other. At 2.30 h, the audience feedback visual feature is 4.99, while the result of the present method is 4.97. This high degree of fit indicates that the method maintains stable accuracy over a short and high span of time.

Figure 5b shows the movie script and line dataset, which contains the script content, character lines, dialog frequency and patterns in the movie. High values represent a heated argument or an important plot twist, while low values indicate a calm dialog or plot transition. The content of the script and the design of the lines can profoundly affect the audience's level of engagement with the story and the characters, and thus the formation of a sense of belonging. Engaging dialog and plots can enhance the audience's sense of agency, while bland scripts alienate the audience. At 2.30 h, audience feedback on movie script and line characteristics was 7.04, while this paper's method yielded a result of 7.03. Effectively capturing the appeal and quality of movie scripts and lines, this accuracy is crucial in enhancing the audience's sense of belonging and immersion.

#### 5.1.3 Comparative analysis of the effects of film music features

**Figure 6:**
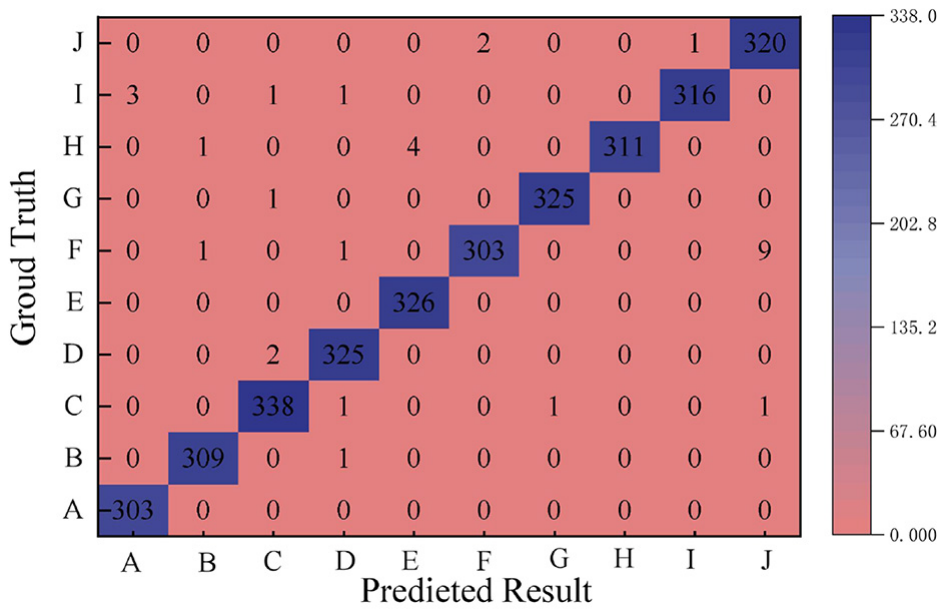
Comparative analysis results of war movie music features.

Figure 6 shows the results of the comparative analysis of the music features of war movies, including 10 musical elements in war movies that can trigger a sense of belonging, A majestic war song, B tragic spin, C war victory theme song, D hometown ballad variations, E emotionally deep strings, F chorus and chorus, G marching style, H patriotic song elements, I reminiscent melody, and J military drums and horns. The X-coordinate represents this paper's algorithm for extracting the war information about the music features of war movies, while the Y coordinate reflects the audience's real understanding feedback about the music features of war movies.

The percentage of the number of correctly recognized picture samples in the dataset of the proposed method is large, which indicates that the proposed method is very effective for music feature recognition. The is-values of the music elements that can trigger a sense of belonging in the 10 war movies are in the range of 303-326, all of which are high, and are analyzed as follows:

(1) The recognition values of the majestic war song A and the chorus and harmony F are both 303, which indicates that these two musical elements occupy a similar position in the audience's real understanding of the music of the war movie, and the model is able to recognize these features better.
(2) Hometown ballad variation D and march style G are 325, which indicates that these elements are also important musical features in war movies that can trigger the audience's sense of belonging. The recognition value of emotionally deep strings E is 326, indicating that emotionally deep strings are a very important feature in war movie music.
(3) Patriotic song element H is 311, I reminiscent melody I is 316, and military drums and horns J are 320, again with relatively high recognition values, suggesting that they are also more prominent features in war movie music. For the mournful melody B is 309, again a common musical element in war movies capable of triggering a sense of belonging. For the war victory theme song C is 338, which has the highest recognition value among the given musical elements, because this musical element is also very typical in war movies In the climax of the movie, the use of a stirring theme song to celebrate the war victory can greatly enhance the audience's sense of belonging and pride in the victory of the country and the nation.

These music elements have an important role in war movies, which can effectively trigger the audience's sense of belonging. And the high recognition value of the model on these elements indicates that the proposed method is effective in music feature recognition.

### 5.2 Analysis of factors influencing viewers' sense of belonging

In studying the influencing factors of viewers' sense of belonging, several dimensions were analyzed, including the degree of viewers' empathy with the characters' emotions, the degree of enhancement of their sense of belonging, as well as viewers' physiological reactions and overall evaluations during the viewing process. This can reveal the level of emotional engagement of the audience and also reflects the potential impact of the attributional characteristics on the audience's sense of belonging.

Table 2 shows the effects of the three features on the audience's sense of belonging, and it can be seen that the three features of character emotions, movie semantics, and music emotions have a significant impact on the audience's sense of belonging. First, in terms of character emotions, the data show that the three emotions of sadness, anger and happiness have a more significant effect on the audience's sense of belonging. The audience empathy level of sadness reached 8.8 points, and the enhancement level of sense of belonging was 8.1 points, indicating that the audience could deeply feel the sadness of the characters and develop a strong sense of belonging. In contrast, although the emotion of fear can also arouse audience empathy, the degree of enhancement of sense of belonging is slightly lower at 6.9 points, while the bland emotion has the worst effect on the enhancement of sense of belonging at 4.7 points. In terms of film semantics, scenes and texts with strong emotional color and story tension are particularly effective in enhancing the audience's sense of belonging. The scene of the defense of hometown has a high level of audience empathy of 9.8 points and a sense of belonging enhancement of 8.7 points, indicating that this kind of scene can stimulate the audience's patriotic feelings and sense of belonging to a high degree. Similarly, the text of comrades' sacrifice and the war victory celebration scene also had a significant effect on the audience's sense of belonging, with a high score of 9.0. However, for the enemy atrocity scene, although it can arouse the audience's attention, the belongingness enhancement effect is 7.0, which is relatively weak. The irrelevant dialog text, on the other hand, has no enhancement effect on the audience's sense of belonging, with a score of only 4.2. In terms of musical emotion, musical elements such as the majestic battle song, the tragic melody, and the variation of the hometown ballad have a very significant enhancement effect on the audience's sense of belonging. For example, the level of audience resonance for the majestic war song reached 9.4 points, and the level of belonging was 8.7 points, showing that this kind of music can greatly enhance the audience's sense of belonging and collective honor. In contrast, the tension and excitement sound effects, although able to attract the audience's attention, had a limited effect on the sense of belonging. Boring background music, on the other hand, has almost no effect on the audience's sense of belonging.

**Table 2:** The impact of three characteristics on audience sense of belonging.

**Feature type**	**Specific feature**	**Audience resonance (1-10)**	**Belongingness improvement (1-10)**	**Physiological index change (increase in heart rate)**	**Audience evaluation (positive/neutral/ negative)**
Character emotion	Sadness	8.8	8.1	+10%	Positive
Character emotion	Anger	7.9	7.3	+12%	Positive
Character emotion	Happiness	8.2	7.6	+8%	Positive
Character emotion	Fear	7.6	6.9	+15%	Neutral
Character emotion	Plain	5.3	4.7	+3%	Negative
Movie semantics	Hometown defense scene	9.8	8.7	+13%	Positive
Movie semantics	Sacrifice of comrades text	9.0	8.5	+11%	Positive
Movie semantics	War victory celebration scene	8.3	7.7	+9%	Positive
Movie semantics	Enemy atrocities scene	7.5	7.0	+14%	Neutral
Movie semantics	Irrelevant dialogue text	4.5	4.1	+2%	Negative
Musical emotion	Majestic war song	9.4	8.7	+12%	Positive
Musical emotion	Solemn melody	8.8	8.3	+10%	Positive
Musical emotion	Variation of hometown folk song	8.5	8.0	+8%	Positive
Musical emotion	Intense and stimulating sound effects	7.4	6.8	+15%	Neutral
Musical emotion	Boring background music	4.9	4.3	+1%	Negative

## 6 Conclusion

This study not only deeply analyzes the multidimensional embodiment of the sense of belonging in war movies from the theoretical level, but also successfully identifies the elements in war movies that are closely related to the sense of belonging through the comprehensive use of multiple methods, including character continuous emotion extraction, movie text feature extraction and music emotion feature extraction. Using pattern recognition techniques such as matrix decomposition, user feature extraction, movie feature extraction and feature fusion, the complex mechanism of sense of belonging construction is further revealed.

The empirical results show that character emotions, movie semantics, and music emotions all have a significant effect on the audience's sense of belonging, in which sadness, anger, and happy emotions trigger a higher degree of audience empathy and sense of belonging enhancement, with ratings of 8.8, 7.9, and 8.2, and 8.1, 7.3, and 7.6, respectively. The hometown defense scene, the text of the sacrifice of comrades, and the victory celebration scene of the war, which are characterized by strongly emotive Semantic elements such as the home defense scene, the sacrifice of comrades and the war victory celebration scene, which have strong emotional colors, can significantly enhance the audience's empathy and sense of belonging, with ratings as high as 9.8, 9.0, 8.3, 8.7, 8.5, 7.7. The influence of musical emotion on the audience's sense of belonging should not be ignored. Infectious musical emotions such as majestic war songs, tragic melodies and variations of hometown ballads can greatly enhance the audience's empathy and sense of belonging, with ratings of 9.2, 8.8, 8.5, and 8.7, 8.3, 8.0, respectively. In the future, the advanced application of algorithms in analyzing the phenomenon of complex emotions can be further explored in order to enhance the effect of the expression of cinematic art and the audience's experience of movie watching.

While the findings of this study shed light on the construction of a media-based sense of belonging in Chinese war films, several limitations should be acknowledged. The present study focused exclusively on popular Chinese war films, selected based on high box office performance and user ratings, which potentially limits the generalizability of the findings to independent productions or films from other cultural and cinematic traditions. This methodological choice aimed to ensure a baseline of public engagement and cultural representativeness within the Chinese media context; however, it may inadvertently omit alternative narrative structures, aesthetic conventions, and thematic representations more common in arthouse or transnational war films (Buolamwini and Gebru, 2018). Therefore, pattern identification derived from mainstream films may reflect dominant discourses of collective belonging, rather than its subversive or marginalized expressions.

In addition, technical limitations must be acknowledged regarding facial expression analysis and audio feature extraction. Facial recognition algorithms are known to be affected by variables such as ethnic morphology, makeup, and lighting environments, which can lead to skewed emotional classification results (Buolamwini and Gebru, 2018). Similarly, soundtrack analysis may be sensitive to mixing techniques, ambient noise, or musical genre conventions, all of which may introduce variation in the semantic-auditory dimension of “belonging” not accounted for in this study.

Theoretically, the study underscores the productive intersection between mediated memory and affective aesthetics in shaping collective identities. Methodologically, it demonstrates how hybrid approaches can bridge quantitative objectivity with qualitative nuance, offering replicable models for future film and media analysis. Lastly, the process of translating affective, subjective audience experiences into quantifiable indices—such as sentiment scores, expression metrics, or clustering outputs—necessitates theoretical caution. While computational tools offer scalable insights into viewer perception, they inevitably risk oversimplifying complex affective phenomena. Thus, the results should be interpreted as indicators of media-encoded belonging, rather than direct psychological proxies.

## Data Availability

The original contributions presented in the study are included in the article/supplementary material, further inquiries can be directed to the corresponding author.
